# Clinical Course and Nutritional Management of Propionic and Methylmalonic Acidemias

**DOI:** 10.1155/2020/8489707

**Published:** 2020-09-16

**Authors:** Amira Mobarak, Heba Dawoud, Hanaa Nofal, Amr Zoair

**Affiliations:** ^1^Pediatrics Department, Faculty of Medicine, Tanta University, Tanta, Egypt; ^2^Clinical Pathology Department, Faculty of Medicine, Tanta University, Tanta, Egypt

## Abstract

Propionic and methylmalonic acidemias result in multiple health problems including increased risk for neurological and intellectual disabilities. Knowledge regarding factors that correlate to poor prognosis and long-term outcomes is still limited. In this study, we aim to provide insight concerning clinical course and long-term complications by identifying possible correlating factors to complications. *Results*. This is a retrospective review of 20 Egyptian patients diagnosed with PA (*n* = 10) and MMA (*n* = 10) in the years 2014–2018. PA patients had lower DQ/IQ and were more liable to hypotonia and developmental delay. The DQ/IQ had a strong negative correlation with length of hospital stay, frequency of PICU admissions, time delay until diagnosis, and the mode ammonia level. However, DQ/IQ did not correlate with age of onset of symptoms or the peak ammonia level at presentation. Both the growth percentiles and albumin levels had a positive correlation with natural protein intake and did not correlate with the total protein intake. Additionally, patients on higher amounts of medical formula did not necessarily show an improvement in the frequency of decompensation episodes. *Conclusion*. Our findings indicate that implementation of NBS, vigilant and proactive management of decompensation episodes, and pursuing normal ammonia levels during monitoring can help patients achieve a better neurological prognosis. Furthermore, patients can have a better outcome on mainly natural protein; medical formula should only be used in cases where patients do not meet 100–120% of their DRI from natural protein.

## 1. Introduction

Propionic acidemia (PA) and methylmalonic aciduria (MMA) are autosomal recessive organic acidemias caused by enzyme defects in the catabolic pathway of propiogenic amino acids. These enzymes are propionyl CoA carboxylase and methylmalonyl CoA, respectively. Despite advances in detection and treatment of these two diseases, the long-term prognosis is still not favourable, especially for patients with propionic acidemia who are at risk for many long-term complications, particularly neurological [1].

As our knowledge about the long-term complications of these two diseases is still limited, our aim for this study is to provide some insight concerning long-term complications in these patient groups through the identification of possible correlating factors to complications. This aim works to contribute to a better understanding of the natural history of these two diseases.

## 2. Patients and Methods

### 2.1. Patients

We reviewed 20 Egyptian patients with PA and MMA from 20 different families diagnosed between the years 2014 and 2018. Details of the study group are shown in Tables 1 and 2. The study was approved by the ethical committee of the Faculty of Medicine, Tanta University, Egypt. Written informed consent was obtained from the patients' parents/legal guardians.

### 2.2. Clinical Examination, Data Collection, and Analysis

Patients' clinical examinations were performed during in-hospital admissions or routine outpatient clinic visits. Data were collected by a retrospective review of medical charts and entered into a Microsoft Office Excel spreadsheet for statistical analysis.

DQ was calculated in patients ≤2 years of age by using the equation DQ = chronological age/developmental age *x* 100, while those between 2 and 7 years had their IQ tested by using the Wechsler Preschool and Primary Scale of Intelligence. Global developmental delay was defined as significant delay (at least 2 SDs below the mean with standardized tests) in at least two developmental domains from the following: gross or fine motor, speech/language, cognition social/personal, and activities of daily living.

Patients were diagnosed with motor delay when they showed unusually slow development of fine motor or gross motor abilities (2SD below the average for sex and age) and were diagnosed with speech delay when either the child's speech was more incoherent than would be expected for age or had speech sound error patterns not appropriate for age.

Data are expressed as mean ± SD, and anthropometric measurements are reported in percentiles. Student's *t*-test was used to compare values of any two independent groups, and Pearson's correlation coefficient was used to evaluate the correlation between independent variables. Significance was set at *p* < 0.05. One-way ANOVA with Bonferroni correction for multiple comparisons was used to compare different growth percentiles with a significance level of *p* < 0.016.

## 3. Results

This study included 20 pediatric Egyptian patients, 10 with PA and 10 with MMA. Patients were diagnosed by selective screening promoted by a positive family history (one patient) or a suspicious clinical presentation (Table 1).

### 3.1. Diet, Physical Development, and Nutritional Status

Average percentile for height/length was 10.85 ± 6.71% with 65% (13/20) ≤ 10^th^ percentile, average weight percentile was 21.25 ± 14.04% with 35% (7/20) ≤ 10^th^ percentile, and head circumference (HC) was 26.25 ± 14.13% with 10% (5/20) on the ≤10^th^ percentile.

**Figure 1:**
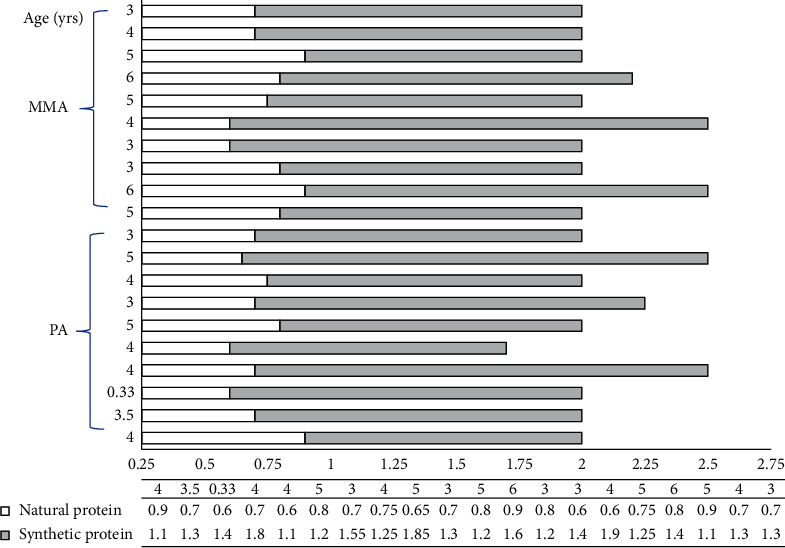
Daily protein intake (gm/kg/day), both intact and synthetic in each individual patient. Patients are categorized by age and diagnosis. The percentage of natural protein intake in all patients was lower than the protein provided through the amino acid formula, 35.23 ± 5.7%, with a range of 24%–45%.

Total protein intake was 2.09 ± 0.24 gm/kg/day, where natural protein intake was 0.73 ± 0.09 gm/kg/day, and protein intake from amino acid formula was 1.37 ± 0.24 gm/kg/day. The ratio between natural and medical formula protein was 0.55 ± 0.13. The percentage of natural protein (35.32 ± 5.75%) was less than that supplied from the medical formula (65.34 ± 5.95%) in all patients, *p* < 0.0001. Total protein intake in all 20 patients was above the recommended Dietary Reference Intakes (DRI). Natural protein intake in 18 patients was within the recommended DRI, with 2 patients below DRI (Figure 1 and Table 3). The number of decompensation episodes did not correlate with total (*r* = −0.14, *p*=0.53), natural (*r* = −0.15, *p*=0.51), or medical formula (*r* = −0.08, *p*=0.72) protein intake.

**Figure 2:**
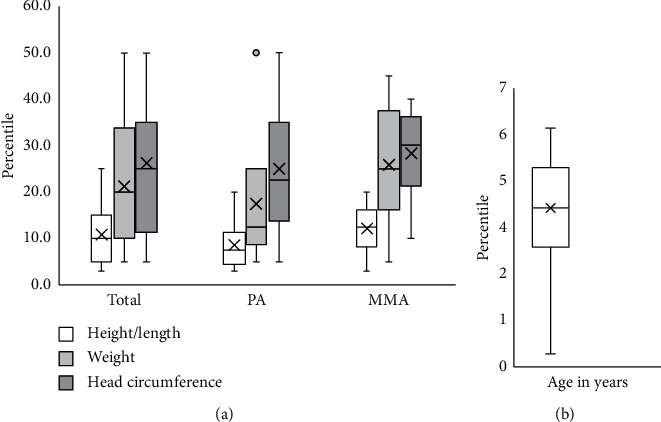
(a) The different growth parameters of the study group, and (b) the age of the study group.

Although our patients were on a high total protein intake, growth parameters were on the lower percentiles with height being the most affected, *p* < 0.0001 (Figure 2). Growth indices (height/length, weight, and head circumference) had a strong positive correlation with both natural protein intake and natural/synthetic protein ratio (Table 4). The two patients whose natural protein intake was below DRI had a height/length on the 3^rd^ percentile, a weight on the 5^th^ percentile, and a HC on the 10^th^ and the 5^th^ percentiles. Growth percentiles did not correlate with total protein or medical formula intake (Table 4), age of onset of symptoms, age at diagnosis, number of metabolic decompensation episodes, number of PICU admissions, and time delay between initial symptoms and diagnosis, *p* > 0.05.

**Figure 3:**
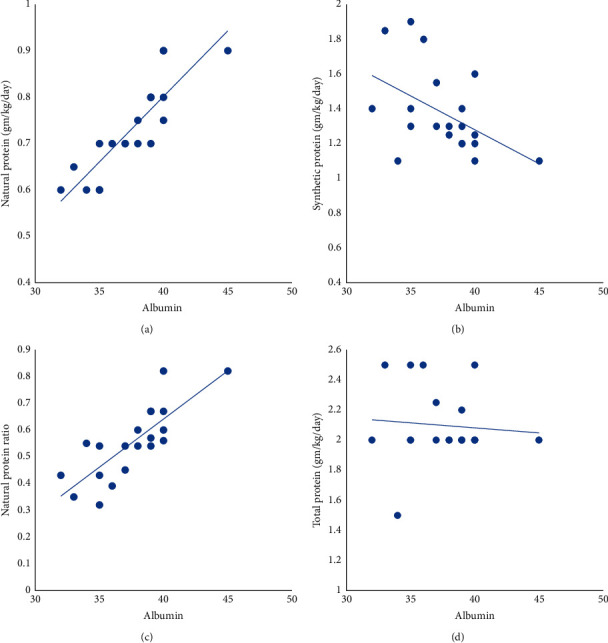
(a) There was a significant positive correlation between the amount of natural protein intake and the albumin level, *r* = 0.87 and *p* < 0.001. (c) There was also a positive correlation between the ratio of natural protein/synthetic protein in diet and the albumin level, *r* = 0.80 and *p* < 0.0001. On the other hand, (b) there was a negative correlation between the synthetic protein intake and the albumin level, *r* = −0.48 and *p*=0.031, and (d) no correlation between the total protein and the albumin level, *r* = −0.08 and *p*=0.73.

Albumin levels measured during periods of metabolic stability were 37.5 ± 3 g/l with no significant difference between MMA and PA patients, *p*=0.35. The albumin level had a significant positive correlation with daily natural protein intake (*r* = 0.8, *p* < 0.001) and natural/synthetic protein ratio *(r* = 0.80, *p* < 0.0001). However, it had a negative correlation with synthetic protein intake (*r* = −0.48, *p*=0.031) and no correlation with total protein intake (*r* = 0.08, *p*=0.82) (Figure 3). Prealbumin levels were 149.55 ± 31.71 mg/l (ref 130–279 mg/l). Unlike albumin levels, prealbumin did not correlate with the natural protein intake (*r* = 0.22, *p*=0.54). The percentage of animal protein in the intake was 23 ± 10.18%, and it had a strong positive correlation with the different growth percentiles, albumin, and prealbumin level (Table 5).

Ionized calcium levels were 1.13 ± 0.08 mmol/l (ref 1.15–1.27 mmol/l), phosphate 1.29 ± 0.22 mmol/l (ref 0.74–1.52 mmol/l), and alkaline phosphatase 134.4 ± 14.59 U/l. Vitamin D levels were 84.41 ± 27.03 nmol/l, with 70% (14/20) of the patients having sufficient levels of >75 nmol/l, 3/20 having levels between 50 nmol/l and < 75 nmol/l, 2/20 having levels between 25 nmol/l and 50 nmol/l, and 1/20 with a level < 25 nmol/l. There was no significant correlation between the protein intake (total, natural, and synthetic) and the level of calcium and vitamin D, *p* > 0.05. Feeding difficulties and nasogastric tube usage were found in 40% (8/20) of the patients, 5 being PA patients.

### 3.2. Neurological Complication

**Figure 4:**
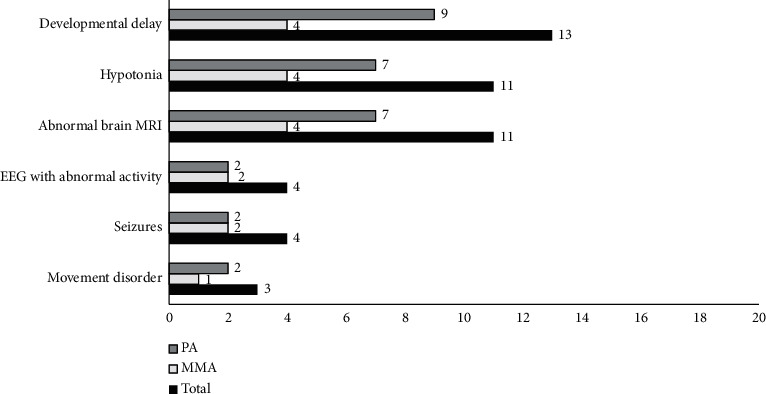
The most frequent neurological complication (*n*).

The most prevalent neurological complication in our cohort was developmental delay in 65% (13/20) of the patients, 4 MMA and 9 PA. Global developmental delay was found in 40%, speech delay in 15%, and motor delay in 15%. The second most encountered complication was hypotonia in 55% (11/20), which was significantly more frequent in PA, *p*=0.038 (Figure 4).

In terms of IQ/DQ, 55% of the patients had an IQ of ≤90. MMA patients tended to have a higher IQ compared to PA patients, *p* < 0.001. There was a significant negative correlation between the IQ/DQ and length of hospital stay, number of metabolic decompensation episodes per year, number of PICU admissions, and the time delay between suspected initial symptoms and diagnosis (3.04 ± 5.53 months). The strongest correlation was with length of hospital stay in the initial episode (5.55 ± 4.36 days) (Tables 6 and 7).

Patients who did not require PICU admission had a higher IQ/DQ (95.44 ± 10.08) when compared to those who did (73.81 ± 23.85, *p*=0.016). In addition, patients with mode ammonia levels of 60 *µ*mol/l (ref 9–33 *µ*mol/l) in monitoring had a significantly lower IQ/DQ when compared to patients whose mode ammonia was <60 *µ*mol/l, *p*=0.03. IQ/DQ had a positive correlation with head circumference (*r* = 0.48, *p*=0.032) but did not correlate with other growth percentiles or protein intake (Table 6).

Abnormal MRI findings were in 55% (11/20) of the patients. Bilateral symmetrical basal ganglia hyperintensity in T2 was noted in 4/11, and increased extra axial spaces, ventricles and prominent brain sulci indicating volume loss were observed in 7/11. Abnormal epileptogenic activity on the EEG accompanied by clinical seizures was found in 4 patients. Three (3/20) patients had movement disorders, two had dystonia and one had transient acute ataxia that was controlled after initiation of treatment (Figure 4).

### 3.3. Clinical Course

In the first year of life, patients had an average of 2.35 ± 1.34 acute metabolic decompensations and then 1.9 ± 1.15 episodes per year thereafter. The length of hospital stay was 5.55 ± 4.35 days during the first episode of decompensation. Eleven (11/20) patients needed PICU admission with an average of 0.9 ± 1.02 admissions per year. PA patients tended to have more frequent metabolic decompensations and a longer length of hospital stay with more frequent PICU admissions (Table 7).

Three (3/20) patients, all PA, died at an average age of 3.25 ± 0.9 years. The cause of death was aspiration pneumonia and respiratory failure in one patient, while the other two patients died of severe sepsis and multiorgan failure.

### 3.4. Hematologic

There was no thrombocytopenia or leucopenia noted outside of the acute decompensation episodes. Hemoglobin ranging between 87 and  < 110 g/l (ref 110–130 g/l) was found in (8/20) patients. The mean ferritin was 33.25 ± 10.26 *µ*g/l (ref 35–350 *µ*g/l) with 9/20 patients having ferritin below 35 *µ*g/l. The ferritin level had a positive correlation with the animal protein portion (Table 5) and did not have a statistically significant correlation with both total (*r* = −0.1; *p*=0.68) and natural dietary protein intake (*r* = 0.38; *p*=0.12).

### 3.5. Other Complications

Six (6/20) patients, including 5 MMA patients, had microalbuminuria of 31.6 ± 1.5 mg/day in a 24-hour urine collection (ref <20 mg) and were started on a low-dose angiotensin-converting enzyme (ACE) inhibitor. Two MMA patients had a GFR of 70 min/1.73 m^2^·min (ref 90–120 mL/min/1.73 m^2^) at the ages of 5 and 6 with a normal pelviabdominal ultrasound. Three PA patients had dilated cardiomyopathy with an average age of 4.5 ± 0.86 at diagnosis.

## 4. Discussion

PA and MMA have a wide spectrum of chronic and subacute complications, with the most devastating ones being neurological [2, 3]. It is essential to identify and ameliorate factors correlating with complications and poor outcome so as to improve medical care and prognosis.

In our cohort, developmental delay was the most frequent complication (*n* = 13) and presented more in PA patients. The second most noted neurological manifestation, which presented significantly more in PA patients, was hypotonia; this is in accordance with other reports where hypotonia was a frequent feature in PA [4].

Patients with PA had a lower IQ/DQ when compared to the MMA group, aligning with previous reports [5, 6], which indicate that PA has unsatisfactory neurological outcomes even when compared to MMA. This can be attributed to our PA patients having a longer length of hospital stay and more frequent decompensation episodes. Nevertheless, our PA patients had a higher IQ/DQ when compared to other published cohorts [7, 8, 9, 10, 11].

Multiple factors correlated with IQ/DQ in our cohort. First, IQ/DQ had a significant negative correlation with the frequency of metabolic decompensation episodes, which is similar to the observations reported by Grunert et al. [7]. In addition, IQ/DQ correlated negatively with length of hospital stay, number of PICU admissions, and time delay between suspected initial symptoms and diagnosis. However, it did not correlate with the age of diagnosis or the age of onset; this is contrary to findings by Surtees et al. [11] who reported that PA patients with early onset had a lower IQ when compared to those with late onset. Usually, patients with early onset tend to have more frequent decompensation episodes that can contribute to cognitive affection, but in our cohort, there was no significant difference between frequency of decompensation episodes in the early-onset (*m* = 1.9 episodes/year) and the late-onset groups (*m* = 1.8 episodes/year) (*p*=0.39). It should be noted that this retrospective study identified age of onset through history taking from caregivers and/or medical records of previous hospital admissions. It is possible that earlier, more subtle symptoms, which may have in fact been more accurate indicators of age of onset, were not recorded through these means; obviously, only major events, such as admissions and ER visits, can be reliably captured, while nonmajor events, which may still cause significant clinical burden, are more difficult to ascertain.

Grunert et al. [7] did not observe a difference in neurocognitive developmental outcomes of a newborn screen (NBS) group. Yet, implementation of NBS along with strict and prompt management of decompensation episodes would help improve neurological outcomes of these patients, especially those who are mild and asymptomatic, as earlier detection and management [10, 12, 13] works to decrease length of hospital stay. This is suggested by our findings that demonstrated a negative correlation between IQ/DQ and length of hospital stay at first decompensation episode and time delay until diagnosis, indicating that rapid successful management of decompensation episodes would contribute to a better neurological outcome. The genetic mutations and the MMA subtype can also play a part in the severity of the clinical phenotype and prognosis. Unfortunately, these data were not available for analysis in our cohort, which is one of the study's limitations.

Ammonia level at presentation did not correlate with IQ/DQ; however, patients who had a mode ammonia level of 60 *µ*mol/l on monitoring, when well, had a significantly lower IQ/DQ. Thus, it is not only the acute elevation of ammonia that has a negative effect on neurocognitive functions but also chronic elevations [14, 15, 16, 17, 18]. This observation supports the hypothesis that using Carbaglu as a chronic medication to normalize ammonia levels can positively impact neurological outcomes [19, 20], but prospective controlled studies are needed to further assert this proposal.

Yannicelli et al. [21] observed improvement in albumin levels six months after starting a group of patients with both MMA and PA on medical formula. They also observed improvement in the anthropometric centiles, therefore concluding that medical formula would enhance the growth of such patients [21]. In our cohort, albumin levels had a negative correlation with medical formula intake and a positive correlation with both natural protein intake and natural/synthetic protein ratio in diet. Unlike albumin, prealbumin did not show a positive correlation to natural protein intake. This can be attributed to the short half-life of prealbumin (2–4 days), making it a better marker for acute changes rather than chronic [22]. Both albumin and prealbumin levels had a positive correlation with the portion of animal protein. It was previously reported that albumin synthesis in healthy men was affected by changes in the proportion of animal versus plant protein in the diet. This can be related to differences in digestibility and consequently in net amino acid availability between diets [23].

Furthermore, despite our patients being on a high amount of medical formula that in turn exceeded the WHO 2007 recommendation for safe protein intake, growth percentiles were on the lower normal, with height/length being most affected. Also, growth percentiles did not correlate with total protein or medical formula intake but did correlate positively with the amount of natural protein in the diet. This aligns with Manoli et al. [24] who did not find a correlation between growth indices and medical formula intake. We also noted that frequency of decompensation episodes did not correlate with protein intake, and patients on higher medical formula intake did not necessarily show more metabolic stability.

Moreover, in a series by G. Touati et al. [25], patient improvement was found to be independent from medical formula and was rather attributed to improvement in management. Management included a low-protein diet based on individual tolerance (versus restricting protein to a set amount given to PKU patients as was performed in the 1970s), a limited use of medical supplements, an overnight enteral feeding with close follow up by a metabolic dietitian, and the use of antibiotics if there was any doubt that the propionic metabolites came mainly from intestinal flora. An Australian study by Evans et al. also showed improvement in MMA/PA patients without the use of medical formula; they have been successfully treating PA/MMA patients without formula since 1991 [26].

Current management uses natural protein (provided that it is tolerated) and adds medical formula when patients fail to reach 100%–120% of the recommended safe protein intake for age and sex [1, 27]. Based on our observations, we are inclined to support the use of natural protein over depending on medical formula, while still maintaining a balance tailored to each patient.

While vegetarian children and adolescents' growth parameters do not differ significantly from nonvegetarians [28], the growth parameters in our cohort of patients correlated positively with the proportion of animal protein given. This can be explained by the fact that vegetarian children are not on a natural protein-restricted diet, making them able to consume larger portions of plant-based protein to compensate for the lesser bioavailability of plant protein. On the other hand, patients with PA and MMA are on a natural protein-restricted diet; therefore, adding an animal protein portion can impact the growth state due to the higher bioavailability in the context of a natural protein-restricted diet.

Regarding bone health parameters, 30% of our cohort had insufficient/deficient vitamin D levels and 90% had mildly decreased calcium. This differed from G. Touati et al. [25] who reported normal bone metabolism markers. It is well known that patients with organic acidemias are liable to bone health disease for multiple reasons, including protein restriction [26] and their dependency on a plant-based diet with low biological value and poor digestibility. However, vitamin D deficiency itself is a well-known nutritional problem among Egyptian children. In a cross-sectional study that involved 200 prepubescent school children aged 9–11, vitamin D deficiency (<20 ng/mL) was detected in 11.5% of subjects, while its insufficiency (levels between 20 and 29.9 ng/mL) was detected in 15%. Obesity, low physical activity, low sun exposure, and low maternal education level were significant predictors of insufficiency, while female gender, low maternal education level, and low milk intake were significant predictors of deficiency [29].

We also noted that 45% of our patients had low ferritin; interestingly, these levels did not correlate with protein intake. However, it did correlate positively with the portion of animal protein intake. This is due to the presence of heme iron in animal protein, which has higher bioavailability compared to nonheme iron from plant sources. Lack of heme iron leads vegetarians to having an iron requirement 1.8 times higher compared to nonvegetarians [30]. These findings indicate that such patients would need early supplementation with micronutrients and vitamins, given that their diet is dependent on medical formula or plant sources, which may not be enough to maintain normal levels of these micronutrients in the long term (Craig 2009; Appleby and Key 2016; Rogerson 2017). A 2010 baseline survey aiming to evaluate the prevalence of iron deficiency anemia in Egypt revealed that ferritin deficiency was most prevalent among mothers (49.6%), followed by adolescents (47.4%), and then preschool- and school-aged children (38.2%). This survey indicated that iron deficiency is a major problem among Egyptian children in general due to dependence on plant-based diets and consumption of iron inhibitors such as phyate, polyphenols, and oxalic acid, revealing a major obstacle to dietary iron consumption and one of the poor dietary habits [31, 32].

## 5. Conclusion

Despite advances in management of both PA and MMA, prognosis—especially neurological—is still unfavourable. Also, massive uniformity of therapeutic interventions hinders our understanding of optimal treatment and our ability to compare patients' outcomes.

The IQ in our cohort correlated negatively with frequency of metabolic crisis, length of hospital stay (first episode and thereafter), and time delay between suspected symptoms and diagnosis initiation. This indicates that the implementation of NBS would improve prognosis by shortening the diagnosis timeframe, allowing for rapid management initiation. The fact that albumin levels and growth percentiles had a positive correlation with natural protein intake, and that patients on high amounts of medical formula did not necessarily show an improvement in frequency of decompensation episodes, suggests that these patients can have a better outcome on mainly natural protein with medical formula only being used when patients fail to meet 100–120% of their RDI from natural protein.

Table 3 shows the daily protein intake of the patients categorized by age group and compared to the PROP recommendations. All patients had a total protein intake (gm/kg/day) that exceeded the recommended DRI for age 2.09 ± 0.24. Two patients had a natural protein intake that is below the DRI while the rest were within recommended levels of intake.

## Figures and Tables

**Table 1:** Summary of the demographic data of the study group.

Demographic data	
Total number of patients	20
Age at the time of data collection (yrs)	3.99 ± 1.29
Time of presentation	
Early onset	12/20 (60%)
Late onset	8/20 (40%)
Diagnosis	
PA	10/20 (50%)
MMA	10/20 (50%)
Ethnic origin	
Middle eastern	20/20 (100%)
Gender	
Female	9/20 (45%)
Male	11/20 (55%)
Family history	
Similar condition	1/20 (5%)
SIDS	3/20 (15%)
Consanguinity	16/20 (80%)
Mode of delivery	
Vaginal	14/20 (70%)
Cs	6/20 (30%)
Gestational age	
Full term	20/20 (100%)

**Table 2:** Comparison between the demographic data of both PA and MMA patients.

Demographic data	PA	MMA
	Number	
Gender		
(i) Female	4 (40%)	5 (50%)
(ii) Male	6 (60%)	5 (50%)
Age of onset		
(i) Early	7 (70%)	5 (50%)
(ii) Late	3 (30%)	5 (50%)
Family history		
(i) Consanguinity	8 (80%)	8 (80%)
(ii) Similar condition	1 (10%)	0
(iii) SIDS	2 (20%)	1 (10%)
Mode of delivery		
(i) C-section	2 (20%)	4 (40%)
(ii) Vaginal delivery	8 (80%)	6 (60%)

**Table 3:** Observed daily protein intake.

Age group	Number	Protein intake (gm/kg/day)				PROP recommendation (gm/kg/day)	
		Intact	Synthetic	Intact (%)	Total	Intact	Total
0–6 months	1	0.6	1.4	30	2	0.91–1.52	1.52–1.82
1–3 years	5	0.7 ± 0.07	1.35 ± 0.13	34 ± 3.5	2.05 ± 0.11	0.63–1.05	1.05–1.26
4–8 years	14	0.75 ± 0.1	1.38 ± 0.28	36 ± 6.3	2.12 ± 0.28	0.57–0.95	0.95–1.14

**Table 4:** Correlation between the different growth percentiles and protein intake.

Growth parameter percentile	Total protein	Natural protein, *r*	Synthetic protein	Natural synthetic protein intake
Height/length	0.06	0.66^*∗∗*^	−0.27	0.5^ǂ^
Weight	0.1	0.56^*∗*^	−0.16	0.4
Head circumference	0.03	0.7^*∗∗∗*^	−0.27	0.64^†^

^*∗*^ *p*=0.01, ^*∗∗*^*p*=0.002, ^*∗∗∗*^*p*=0.001, ^†^*p*=0.002 and ^ǂ^*p*=0.024.

**Table 5:** Correlation between the percentage of animal protein intake and different growth and nutritional parameters.

	Height/Length	Weight	Head circumference	Albumin	Prealbumin	Vitamin D	Ferritin
	*r*						
Prealbumin	0.37	0.39	0.61^*∗*^	0.91112^*∗*^			
Animal protein percentage	0.71^*∗*^	0.76^*∗*^	0.67^*∗*^	0.57^*∗*^	0.45^*∗∗*^	0.49^*∗∗∗*^	0.51^*∗*^

^*∗*^ *p* < 0.01, ^*∗∗*^*p*=0.04, and ^*∗∗∗*^*p*=0.02.

**Table 6:** Correlation between IQ/DQ and the clinical course, growth centiles, nutrition, and biochemical investigations.

	IQ/DQ level	
(1) Clinical course	*r*	*p* value
(i) Time delay between first symptoms and diagnosis	−0.51	0.021^*∗*^
(ii) Age of diagnosis	−0.08	0.74
(iii) Number of attacks in first year	−0.039	0.088
(iv) Number of attacks per year	−0.53	0.015^*∗*^
(v) Length of hospital stay in first attack	−0.86	<0.001^*∗*^
(vi) Number of ICU admissions	−0.66	0.002
(vii) Length of hospital stay	−0.87	<0.00001^*∗*^

(2) Nutritional status and growth percentiles		
(i) Total protein intake	−0.03	0.2
(ii) Natural protein intake	0.3	0.2
(iii) Synthetic protein intake	−0.16	0.5
(iv) Natural to synthetic protein ratio	0.31	0.18
(v) Length/height	0.43	0.06
(vi) Weight	0.37	0.1
(vi) Head circumference	0.48	0.032^*∗*^

(3) Biochemical investigations in the first attack		
(i) Ammonia	−0.25	0.28
(ii) Lactate	−0.13	0.57
(iii) Bicarbonate	−0.02	0.93
(iv) pH	0.1	0.66
(v) C3	−0.2	0.38
(vi) C3: C2	0.21	0.37
(vii) Glycine	−0.2	0.39

^*∗*^ Statistical significance with *p* < 0.05.

**Table 7:** Comparison between PA and MMA groups.

Variable	Total	PA	MMA	*p* value
	Mean ± SD			
Dietary intake (gm/kg/day)				
(i) Total protein	2.097 ± 0.24	2.075 ± 0.28	2.12 ± 0.2	0.69
(ii) Natural protein	0.73 ± 0.99	0.71 ± 0.09	0.75 ± 0.1	0.32
(iii) Synthetic amino acid formula	1.37 ± 0.24	1.38 ± 0.26	1.36 ± 0.23	0.86
(iv) Natural protein: synthetic protein	0.55 ± 0.13	0.53 ± 0.13	0.55 ± 0.13	0.54
Anthropometric measures (percentiles)				
(i) Length/height	10.85 ± 6.71	8.6 ± 5.56	13.1 ± 7.27	0.13
(ii) Head circumference	26.25 ± 14.13	25 ± 15.09	27.5 ± 13.79	0.7
(iii) Weight	21.25 ± 14.03	17.5 ± 13.59	25 ± 14.14	0.24
Clinical course				
(i) Length of hospital stay^*∗*^	5.55 ± 4.35	8.4 ± 4.4.59	0.33 ± 0.5	0.003^*∗*^
(ii) Length of hospital stay^*∗∗*^	6.1 ± 0.65	7.9 ± 3.1	4.3 ± 1.15	0.005^*∗*^
(iii) Decompensation episodes per year	1.9 ± 1.15	2.6 ± 1.07	1 ± 0.43	0.003^*∗*^
(iv) PICU admission frequency	0.9 ± 1.02	1.3 ± 1.15	0.33 ± 0.5	0.04^*∗*^
(v) Attacks in the first year	2.32 ± 1.34	2.8 ± 1.47	1.6 ± 0.8	0.14
(vi) IQ/DQ	83. ± 0.24	70 ± 23.09	97.1 ± 6.22	0.004^*∗*^

^*∗*^During the initial decompensation episode and ^*∗∗*^after the initial episode.

## Data Availability

The data used to support this study are included within the manuscript.
